# Analysis of total corneal astigmatism with a rotating Scheimpflug camera in keratoconus

**DOI:** 10.1186/s12886-020-01747-9

**Published:** 2020-12-03

**Authors:** Jinho Kim, Woong-Joo Whang, Hyun-Seung Kim

**Affiliations:** 1 Department of Ophthalmology, Seoul St. Mary’s Hospital, College of Medicine, The Catholic University of Korea, Seoul, South Korea; 2 Department of Ophthalmology, Yeouido St. Mary’s Hospital, College of Medicine, The Catholic University of Korea Korea, Seoul, South Korea

## Abstract

**Background:**

To analyze mean corneal powers and astigmatisms on anterior, posterior, and total cornea in patients with keratoconus as calculated according to various keratometric measurements using a Scheimpflug camera.

**Methods:**

We examined the left eyes of 64 patients (41 males and 23 females; mean age 29.94 ± 6.63 years) with keratoconus. We measured simulated K (Sim-K), posterior K, true net power (TNP) and four types of total corneal refractive powers (TCRP). We then used the obtained values to analyze mean K, and corneal astigmatism. TCRP were measured at 2.0 ~ 5.0 mm.

**Results:**

Mean corneal powers from Sim K, posterior K, and TNP were 49.12 ± 3.99, − 7.39 ± 0.79, and 47.78 ± 4.09 diopters, respectively. For TCRP centered on the pupil, mean K tended to decrease with measurement area (all *p* < 0.01). While, both mean K and astigmatism measured using TCRP centered on the apex decreased with measurement area (all *p* < 0.001). TCRP centered on the apex were greater than those centered on the pupil for mean K values calculated using TCRP (all *p* < 0.001). The proportion of WTR was greatest on the anterior and total cornea. As the measurement area moved to the periphery, the proportion of WTR increased.

**Conclusions:**

Mean corneal powers and astigmatisms on total cornea with keratoconus change depending on calculation methods and measurement areas.

## Background

Keratoconus is a progressive non-inflammatory disease characterized by thinning and protrusion of the cornea, resulting in high degrees of irregular astigmatism and myopia that lead to impairment of visual quality and distorted vision [1].

The Pentacam® is a rotating Scheimpflug camera used to evaluate the topography of the corneal surface and measure corneal thickness, which may be helpful in the diagnosis of keratoconus and identification of disease stage [2]. Although corneal power has traditionally been assessed using instruments that measure anterior corneal power alone, a rotating Scheimpflug camera has made it possible to measure posterior corneal curvature as well [3]. Furthermore, keratometric and pachymetric measurements by a Scheimpflug rotating camera were repeatable than placido topographer combined with slit-scanning technology [3].

Kamiya et al. [4] investigated eyes with keratoconus and concluded that 78.8% exhibit ATR astigmatism, while only 10.2 and 10.9% exhibit with-the-rule (WTR) and oblique astigmatism, respectively. The authors of the aforementioned study further reported a mean magnitude of posterior corneal astigmatism in keratoconus of 0.93 diopters. Naderan et al. [5] also reported a similar value for the magnitude of posterior corneal astigmatism in keratoconic eyes (0.90 diopters), which is far greater than the magnitude observed in normal eyes (0.26 to 0.78 D) [6–12]. In contrast with mean magnitude of posterior corneal astigmatism, WTR astigmatism was more prevalent than oblique and ATR astigmatism in their study.

Keratometric measurements taking into account only the anterior corneal power utilize a corneal index of refraction of 1.3375. This assumption is derived from the concept that the posterior radius of curvature is 1.2 mm steeper than the anterior corneal radius of curvature [13]. However, this value is not consistent in eyes with keratoconus, in which the relationship between the anterior and posterior corneal radii has become distorted [14–16]. Therefore, use of 1.3375 as the keratometric index in patients with keratoconus is imprecise and may result in an overestimation of corneal power [17]. Camps et al. [18] calculated an adjusted keratometric index ranging from 1.3190 to 1.3324 using the Gullstrand eye model, though such a value would be affected by the degree of disease progression. Watson et al. [19] further identified overestimation of corneal power as the primary cause of postoperative hyperopic prediction error when a conventional keratometer was used.

Purpose of this study is to analyze the various corneal measurements including Simulated K, posterior K, true net power (TNP), and 4 types of total corneal refractive power (TCRP) obtained from the eyes with keratoconus using the Pentacam® rotating Scheimpflug camera and to evaluate changes in mean corneal power and corneal astigmatism due to measurement method and area.

## Methods

### Patients and study design

In the present retrospective study, we analyzed the left eyes of 64 patients (41 males and 23 females) who had been diagnosed with keratoconus between Jan 2017 and Jan 2019. The Institutional Review Board for Human studies at Yeouido St. Mary Hospital (Seoul, Korea) reviewed and approved this study protocol (SC19RESI0111). As this study was a retrospective study, verbal informed consent was obtained from all patients before beginning data collection and analyses. All study conduct adhered to the tenets of the Declaration of Helsinki for the use of human participants in biomedical research.

Diagnoses of keratoconus were confirmed by an experienced clinician (W.J.W.) based on slit-lamp observation and measurements obtained using a Scheimpflug rotating camera. Characteristic features of keratoconus were confirmed in all cases: asymmetric bow-tie pattern with/without skewed axis, Fleischer rings, or Vogt’s striae. They were also confirmed by the Amsler-Krumeich classification based on corneal astigmatism, corneal power, corneal transparency, and corneal thickness [20]. Patients with visually significant cataracts, corneal scarring, iris abnormalities, history of glaucoma or retinal disease, macular disease, retinopathy, neuro-ophthalmic disease, history of ocular inflammation, or previous ocular surgeries were excluded.

Patients were instructed to discontinue use of rigid gas permeable (RGP) or soft contact lenses for 3 weeks, following which imaging with the Pentacam® rotating Scheimpflug camera (Oculus; Wetzler, Germany) was performed. A 25-picture scan was used examine each cornea, and only scans graded as being “OK” according to instrument specifications were included in this study. One skilled operator (W.J.W) obtained three measurements and analyzed the average value from three measurements.

Total corneal power is calculated based on anterior corneal power, posterior corneal power, and corneal thickness. Both the Gaussian optic formula and ray-tracing method are applied when calculating total corneal power. Furthermore, total corneal power can be calculated for each zone or ring. Consequently, currently available Scheimpflug cameras allow one to investigate almost 40 combinations of corneal power [20].

#### Simulated K (Sim-K)

The Sim-K value represents the mean corneal power calculated by simulated keratometry and is the arithmetic mean of a pair of meridians spaced 90 degrees apart, with the greatest difference in axial power lying within a central 3.0 mm zone. Sim-K is calculated by entering the corneal curvature radius into a thin-lens formula for paraxial imagery, which considers the cornea as a single refractive sphere. The cornea radii are converted into dioptric power values using the keratometric index of refraction (1.3375).

#### True net power (TNP)

The True Net Power (TNP) represent the optical power of the cornea based on two different refractive indices: one for the anterior surface (corneal tissue: 1.376) and one for the posterior surface (aqueous humor: 1.336). TNP is calculated using a Gaussian optic formula that also takes into account the sagittal curvature of each surface.

#### Total corneal refractive power (TCRP)

The Total Corneal Refractive Power (TCRP) value is automatically measured according to the ray-tracing method. TCRP is calculated using the values for anterior radius, posterior radius, and corneal thickness. Snell’s law and the specific refractive indices of air, cornea, and aqueous humor are used to calculate the corneal power, resulting in four types of TCRP measurements: (1) *Pupil (zone*), corneal power centered on the pupil and measured over the inner zone; (2) *Pupil (ring*), corneal power centered on the pupil and measured over a ring; (3) *Apex (zone)*, corneal power centered on the apex and measured over the inner zone; (4) *Apex (ring)*, corneal power centered on the apex and measured over a ring.

### Assessment of keratometric measurement and statistical analysis

From the above measurements, we calculated the flattest keratometric value (flat K), steepest keratometric value (steep K), mean keratometric value (mean K) in order to assess overall types and degrees of astigmatism. TCRP values were measured at 2.0 mm, 3.0 mm, 4.0 mm, and 5.0 mm. We also divided total 64 eyes into two groups (28 eyes with stage 1 and 36 eyes with stage 2 ~ 4) according to Amsler-Krumeich classification and calculated corneal refractive power [20]. All types of astigmatism except that of the posterior cornea were classified as with-the-rule (WTR) when the steep meridian was within the range of 60–120 degrees and against-the-rule (ATR) when the steep meridian was within the range of either 150–180 degrees or 0–30 degrees. The remaining instances were classified into oblique astigmatism (steep meridian ranging from 30 to 60 degrees and from 120 to 150 degrees). This classification was possible due to the inclusion of left eyes only. Posterior corneal astigmatism was classified as WTR when the steep meridian was within the range of either 150–180 degrees or 0–30 degrees, and as ATR when the steep meridian was within the range of 60–120 degrees. The remaining instances were classified into oblique astigmatism. A net astigmatism is given as (M@ *α*), where M is the astigmatic magnitude in diopters (D) and *α* is the astigmatic direction in degrees [21].

$$ \mathrm{Polar}\ \mathrm{value}\ \mathrm{along}\ \mathrm{the}\ \mathrm{zero}\ \mathrm{degree}\ \mathrm{meridian}=\mathrm{KP}\ (0)=\mathrm{M}\ast \mathit{\cos}\left(2\ast a\right) $$

$$ \mathrm{Polar}\ \mathrm{value}\ \mathrm{along}\ \mathrm{the}\ 45\ \mathrm{degrees}\ \mathrm{meridian}=\mathrm{KP}\ (45)=\mathrm{M}\ast \mathit{\sin}\left(2\ast a\right) $$

Additionally, the axis and magnitude of astigmatism were represented using a double-angle polar plot (Astig PLOT) in Eye Pro 2013 (for iPhone/iPad; Apple; Cupertino, California, USA) developed by Dr. Edmondo Borasio. All statistical analyses were performed using IBM SPSS Statistics for Windows, Version 21.0 (IBM Corp, Armonk, NY, USA). The sample size of this study was sufficient to offer a power of 95% statistical power at a significance level of 5%, and detected 1.0 diopter difference of corneal refractive power by G*power (Version 3.1.9.6, https://www.gpower.hhu.de). All *p*-values ≤0.05 were considered statistically significant. Friedman tests were performed to determine the differences due to measurement area and Wilcoxon signed ranked tests were performed to determine differences between total corneal refractive power centered on the pupil and apex.

## Results

A total 64 left eyes (64 patients) were evaluated in the present study. The mean age was 29.94 ± 6.63 years (range: 18–44 years) and the two subgroups showed no significant difference in age (29.81 ± 6.34 years with stage 1 keratoconus and 30.03 ± 6.93 years with stage 2 ~ 4 keratoconus: *p* = 0.76). The demographic characteristics of the included patients are summarized in Table 1. Mean corneal power for the anterior and posterior corneal surfaces were 49.12 ± 3.99 diopters and − 7.39 ± 0.79 diopters, respectively. Mean corneal power from TNP was 47.78 ± 4.09 diopters. Statistically significant difference was observed in mean K values between measurements of anterior corneal power and true net power (*p* < 0.001). No significant difference was observed between astigmatism derived from true net power and that derived from power of the anterior surface (*p* = 0.34).

**Table 1:** Simulated K, posterior K, and true net power (TNP)

	Simulated K	Posterior K	True net power (TNP)	* *p* value
Mean keratometry (diopter)	49.12 ± 3.99	−7.39 ± 0.79	47.78 ± 4.09	< 0.001
Mean arithmetic astigmatism (diopter)	4.58 ± 2.14	0.91 ± 0.47	4.59 ± 2.17	0.34
KP(0) (diopter)	− 2.74 ± 3.24	0.64 ± 0.61	− 2.78 ± 3.31	0.81
KP(45) (diopter)	1.40 ± 2.40	−0.27 ± 0.45	1.25 ± 2.39	< 0.001

^*^The Wilcoxon signed ranked test was used in comparison of simulated K and true net power

TCRP values for the 2.0–5.0 mm zones are listed in Table 2. As the measurement zone expanded, mean K tended to decrease in TCRP when centered on pupil (*p* = 0.005, respectively). Mean K, and corneal astigmatism provided by TCRP centered on apex significantly decreased according to changes in measurement zone (all *p* < 0.001). TCRP centered on the apex resulted in greater values than TCRP centered on the pupil for all measurements, with the exception of corneal astigmatism at the 4.0 mm and 5.0 mm zones. Changes in corneal power according to measurement zone were also greater in TCRP centered on apex. Table 3 demonstrates TCRP values for the 2.0–5.0 mm zone in two subgroups. There was no difference in mean K with stage 1 (*p* > 0.05). However, in stage 2 ~ 4, TCRP significantly decreased as the measurement area widened (all *p* < 0.001).

**Table 2:** Mean arithmetic values and power vectors for total corneal refractive power within 2.0–5.0 mm zones

		2.0 mm zone	3.0 mm zone	4.0 mm zone	5.0 mm zone	* *p* value
Centered on pupil	Mean keratometry (diopter)	48.28 ± 5.19	47.96 ± 4.53	47.62 ± 3.83	47.20 ± 3.15	0.005
	Mean arithmetic astigmatism (diopter)	3.62 ± 2.50	4.32 ± 2.11	4.52 ± 1.99	4.41 ± 1.97	< 0.001
	KP(0) (diopter)	− 1.70 ± 2.72	− 2.42 ± 3.09	− 2.83 ± 3.23	− 2.99 ± 3.13	< 0.001
	KP(45) (diopter)	1.44 ± 2.66	1.38 ± 2.45	1.21 ± 2.16	1.05 ± 1.91	0.17
Centered on apex	Mean keratometry (diopter)	49.83 ± 5.92	49.29 ± 5.16	48.68 ± 4.31	48.03 ± 3.99	< 0.001
	Mean arithmetic astigmatism (diopter)	5.35 ± 3.16	5.02 ± 2.66	4.61 ± 2.16	4.15 ± 1.81	< 0.001
	KP(0) (diopter)	−2.64 ± 4.15	−2.70 ± 3.75	−2.68 ± 3.30	−2.59 ± 2.87	0.32
	KP(45) (diopter)	1.72 ± 3.43	1.53 ± 2.97	1.35 ± 2.49	1.12 ± 2.12	0.001
** *p* value for mean keratometry		< 0.001	< 0.001	< 0.001	< 0.001	
** *p* value for mean arithmetic astigmatism		< 0.001	< 0.001	0.62	0.07	
** *p* value for KP(0)		0.025	0.60	0.19	0.002	
** *p* value for KP(45)		0.011	0.076	0.068	0.36	

*Friedman test was used in comparison of keratometric values according to measurement zone

** Wilcoxon signed ranked test was used in comparison of keratometric values centered on pupil versus apex

**Table 3:** Mean arithmetic values and power vectors for total corneal refractive power within 2.0–5.0 mm zones

		Stage 1					Stage 2 ~ 4				
		2.0 mm zone	3.0 mm zone	4.0 mm zone	5.0 mm zone	* *p* value	2.0 mm zone	3.0 mm zone	4.0 mm zone	5.0 mm zone	* *p* value
Centered on pupil	Mean keratometry (diopter)	44.43 ± 1.53	44.52 ± 1.38	44.66 ± 1.25	44.76 ± 1.15	0.06	49.81 ± 4.87	49.31 ± 4.25	48.75 ± 3.58	48.12 ± 2.97	< 0.001
	Mean arithmetic astigmatism (diopter)	2.33 ± 1.04	2.72 ± 0.95	2.84 ± 1.07	2.86 ± 1.23	0.001	4.44 ± 2.62	5.07 ± 2.23	5.15 ± 2.05	4.92 ± 2.10	0.037
	KP(0) (diopter)	−1.03 ± 1.79	− 1.49 ± 1.90	−1.82 ± 1.91	−2.08 ± 1.82	< 0.001	− 2.44 ± 3.20	− 3.05 ± 3.53	− 3.32 ± 3.58	− 3.34 ± 3.51	< 0.001
	KP(45) (diopter)	0.89 ± 1.27	1.02 ± 1.27	0.99 ± 1.20	0.97 ± 1.11	0.012	1.48 ± 2.92	1.33 ± 2.75	1.04 ± 2.49	0.77 ± 2.21	0.002
Centered on apex	Mean keratometry (diopter)	45.07 ± 1.55	45.08 ± 1.40	45.12 ± 1.28	45.14 ± 1.21	0.92	51.41 ± 5.56	50.66 ± 4.81	49.83 ± 3.99	48.94 ± 3.21	< 0.001
	Mean arithmetic astigmatism (diopter)	3.09 ± 1.55	3.07 ± 1.25	3.02 ± 1.10	2.90 ± 1.12	0.93	6.13 ± 2.88	5.69 ± 2.52	5.12 ± 2.24	4.54 ± 2.05	< 0.001
	KP(0) (diopter)	−1.37 ± 2.52	−1.57 ± 2.33	−1.81 ± 2.16	−1.94 ± 1.99	0.23	− 3.52 ± 4.35	−3.37 ± 3.97	−3.14 ± 3.59	−2.85 ± 3.24	0.68
	KP(45) (diopter)	1.22 ± 1.57	1.16 ± 1.40	1.04 ± 1.24	0.92 ± 1.10	0.18	1.91 ± 3.39	1.64 ± 3.06	1.37 ± 2.64	1.11 ± 2.29	0.021
** *p* value for flat keratometry		0.041	< 0.001	< 0.001	< 0.001		0.002	< 0.001	< 0.001	< 0.001	
** *p* value for mean arithmetic astigmatism		< 0.001	0.004	0.054	0.76		< 0.001	0.001	0.98	0.076	
** *p* value for KP(0)		0.091	0.72	0.95	0.046		0.011	0.094	0.13	0.003	
** *p* value for KP(45)		0.022	0.44	0.80	0.55		0.023	0.016	0.006	0.006	

*Friedman test was used in comparison of keratometric values according to measurement zone

** Wilcoxon signed ranked test was used in comparison of keratometric values centered on pupil versus apex

Table 4 indicates the dioptric power values of TCRPs in the 2.0–5.0 mm rings, which were similar to those obtained for the 2.0–5.0 mm zones. Statistically significant differences were observed for all dioptric powers between measurement rings (all *p* < 0.001) and for all values with the exception of corneal astigmatism calculated according to TCRP centered on the pupil, which tended to decrease as the measurement ring extended to the periphery. TCRP calculation centered on the apex resulted in greater refractive power values for mean K and corneal astigmatism at the 2.0 mm ring, while TCRP calculation centered on the pupil resulted in greater values for corneal astigmatism at 3.0–5.0 mm rings (all *p* < 0.05). TCRP values for the 2.0–5.0 mm ring in two subgroups are listed in Table 5. For mean arithmetic corneal astigmatism in TCRP centered on pupil and mean K in TCRP centered on apex, stage 1 keratoconus showed no statistical difference (all *p* > 0.05) and stage 2 ~ 4 keratoconus showed significant difference (all *p < 0.001).*

**Table 4:** Mean arithmetic values and power vectors for total corneal refractive power at 2.0–5.0 mm rings

		2.0 mm ring	3.0 mm ring	4.0 mm ring	5.0 mm ring	*p value
Centered on pupil	Mean keratometry (diopter)	47.98 ± 4.59	47.47 ± 3.54	46.86 ± 2.51	46.15 ± 1.91	< 0.001
	Mean arithmetic astigmatism (diopter)	4.77 ± 2.28	4.99 ± 2.23	4.81 ± 2.33	4.07 ± 2.30	< 0.001
	KP(0) (diopter)	− 2.65 ± 3.42	− 3.23 ± 3.64	−3.45 ± 3.51	− 3.08 ± 3.00	0.001
	KP(45) (diopter)	1.51 ± 2.68	1.18 ± 2.22	0.87 ± 1.95	0.69 ± 1.74	0.002
Centered on apex	Mean keratometry (diopter)	49.39 ± 5.27	48.42 ± 3.96	47.40 ± 2.70	46.48 ± 1.77	< 0.001
	Mean arithmetic astigmatism (diopter)	5.19 ± 2.79	4.55 ± 2.09	4.04 ± 1.72	3.28 ± 1.55	< 0.001
	KP(0) (diopter)	−2.73 ± 3.91	−2.70 ± 3.26	−2.58 ± 2.79	−2.29 ± 2.25	0.081
	KP(45) (diopter)	1.64 ± 3.09	1.20 ± 2.42	0.86 ± 2.06	0.65 ± 1.59	< 0.001
** *p* value for mean keratometry		< 0.001	< 0.001	< 0.001	< 0.001	
** *p* value for mean arithmetic astigmatism		0.002	0.01	< 0.001	< 0.001	
** *p* value for KP(0)		0.66	< 0.001	< 0.001	< 0.001	
** *p* value for KP(45)		0.041	0.78	0.57	0.18	

*Friedman test was used in comparison of keratometric values according to measurement ring

** Wilcoxon signed ranked test was used in comparison of keratometric values centered on pupil versus apex

**Table 5:** Mean arithmetic values and power vectors for total corneal refractive power at 2.0–5.0 mm rings

		Stage 1					Stage 2 ~ 4				
		2.0 mm ring	3.0 mm ring	4.0 mm ring	5.0 mm ring	*p value	2.0 mm ring	3.0 mm ring	4.0 mm ring	5.0 mm ring	*p value
Centered on pupil	Mean keratometry (diopter)	44.51 ± 1.41	44.70 ± 1.21	44.88 ± 1.10	45.00 ± 1.06	< 0.001	49.35 ± 4.30	48.52 ± 3.31	47.56 ± 2.38	46.54 ± 2.06	< 0.001
	Mean arithmetic astigmatism (diopter)	2.96 ± 1.07	3.10 ± 1.26	3.15 ± 1.47	2.96 ± 1.46	0.20	5.58 ± 2.31	5.55 ± 2.30	5.16 ± 2.44	4.19 ± 2.49	< 0.001
	KP(0) (diopter)	−1.58 ± 2.12	−2.09 ± 2.05	− 2.45 ± 1.99	−2.51 ± 1.75	0.10	− 3.35 ± 3.85	− 3.67 ± 3.98	− 3.65 ± 3.81	−3.00 ± 3.36	0.15
	KP(45) (diopter)	1.11 ± 1.37	1.09 ± 1.26	0.98 ± 1.13	0.85 ± 0.92	0.44	1.51 ± 2.94	0.91 ± 2.53	0.46 ± 2.20	0.26 ± 1.92	0.001
Centered on apex	Mean keratometry (diopter)	45.09 ± 1.43	45.13 ± 1.26	45.22 ± 1.11	45.17 ± 1.15	0.97	50.80 ± 4.91	49.45 ± 3.67	48.06 ± 2.46	46.86 ± 1.64	< 0.001
	Mean arithmetic astigmatism (diopter)	3.15 ± 1.28	3.09 ± 1.12	3.00 ± 1.31	2.66 ± 1.31	0.005	5.86 ± 2.66	4.94 ± 2.30	4.22 ± 2.14	3.43 ± 1.88	< 0.001
	KP(0) (diopter)	−1.56 ± 2.42	−1.92 ± 2.20	−2.20 ± 2.07	−2.18 ± 1.73	0.16	− 3.44 ± 4.13	−3.04 ± 3.55	− 2.61 ± 3.22	−2.14 ± 2.77	0.32
	KP(45) (diopter)	1.20 ± 1.45	0.98 ± 1.21	0.79 ± 1.06	0.62 ± 0.89	0.12	1.75 ± 3.16	1.19 ± 2.62	0.79 ± 2.20	0.57 ± 1.70	0.002
** *p* value for flat keratometry		< 0.001	< 0.001	< 0.001	< 0.001		< 0.001	< 0.001	< 0.001	< 0.001	
** *p* value for steep keratometry		< 0.001	< 0.001	0.10	0.78		< 0.001	< 0.001	0.25	0.017	
** *p* value for mean keratometry		< 0.001	< 0.001	< 0.001	< 0.001		< 0.001	< 0.001	< 0.001	< 0.001	
** *p* value for mean arithmetic astigmatism		0.022	0.99	0.06	0.004		0.032	0.006	< 0.001	< 0.001	
** *p* value for KP(0)		0.63	0.030	0.003	< 0.001		0.60	< 0.001	< 0.001	< 0.001	
** *p* value for KP(45)		0.52	0.050	0.005	0.002		0.009	0.024	0.020	0.076	

*Friedman test was used in comparison of keratometric values according to measurement ring

** Wilcoxon signed ranked test was used in comparison of keratometric values centered on pupil versus apex

**Fig. 1:**
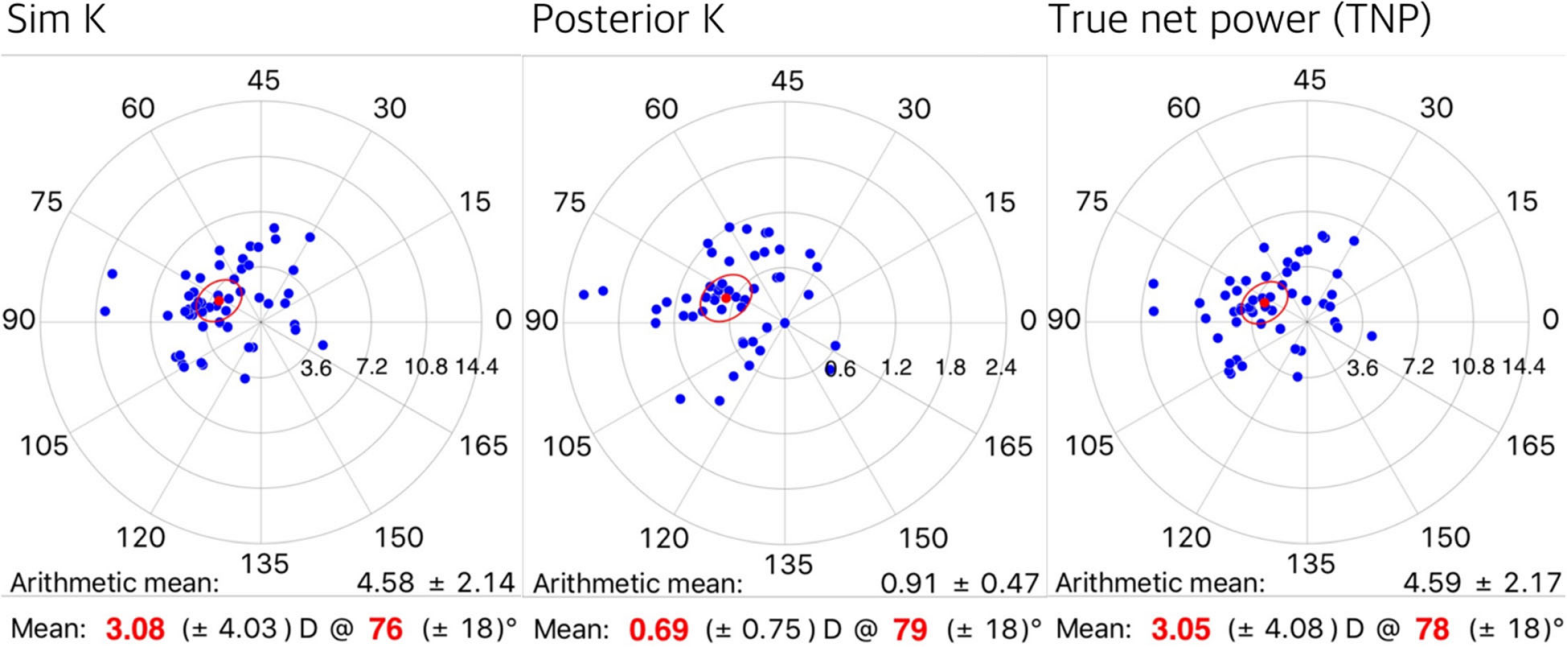
Mean corneal astigmatisms represented on double-angle polar plots by simulated K, posterior K, and true net power

**Fig. 2:**
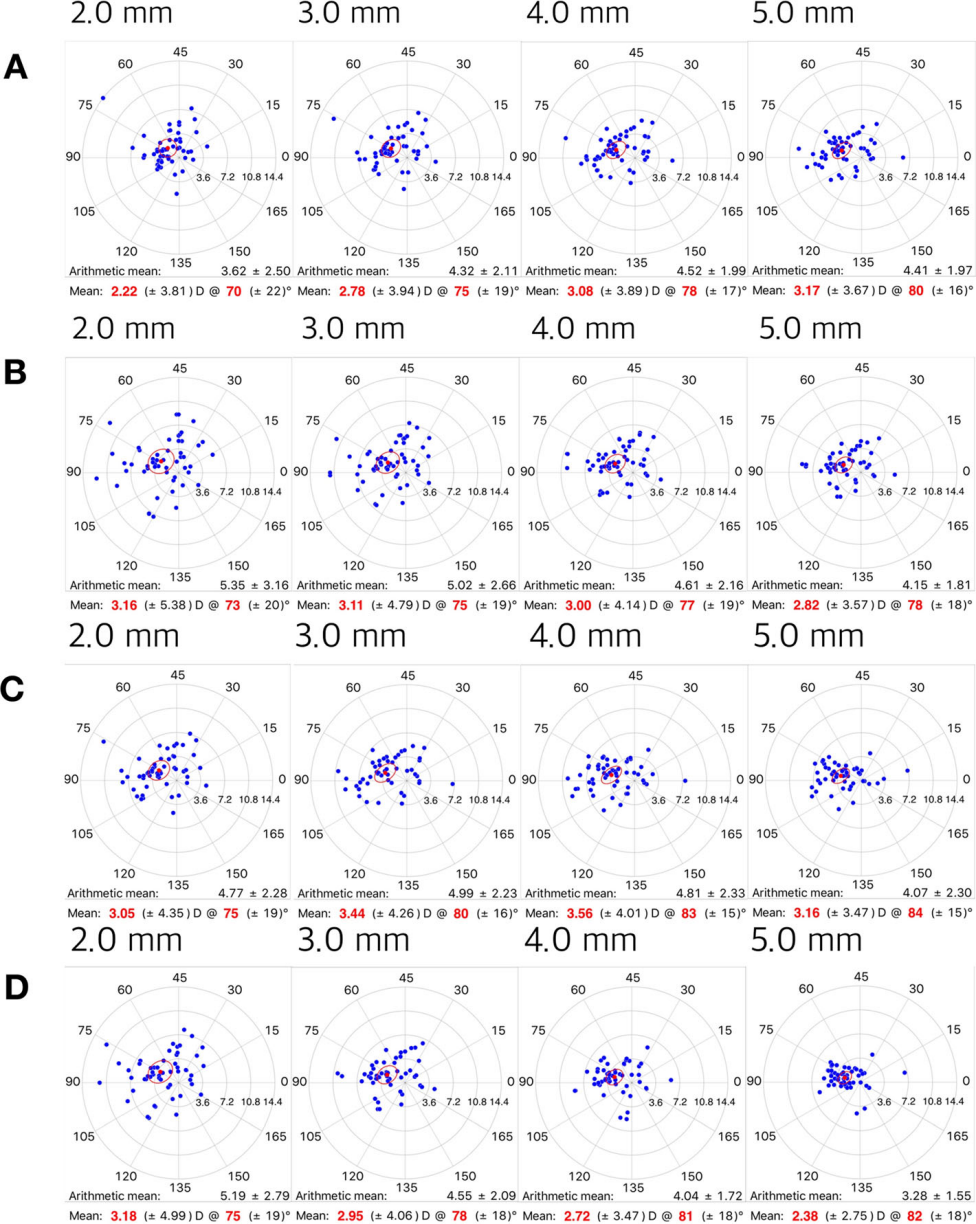
Mean corneal astigmatisms represented on double-angle polar plots by total corneal refractive power (TCRP). **a** 2.0 mm–5.0 mm zone centered on the pupil; (**b**) 2.0 mm–5.0 mm zone centered on the apex; (**c**) 2.0 mm–5.0 mm ring centered on the pupil; (**d**) 2.0 mm–5.0 mm ring centered on the apex

Figures 1 and 2 depict mean corneal astigmatism values on a double-angle plot. The steep axes for anterior and posterior K were located at 76 and 79 degrees, respectively; while the steep axis for TNP was located at 78 degrees. As the measurement area shifted toward the periphery, the steep axis shifted toward WTR astigmatism for TCRP measurements. The magnitude of mean corneal astigmatism as calculated by TCRP increased with more peripheral measurement for pupil-centered zones, while the same value decreased with more peripheral measurement for TCRP apex-centered zones and rings.

**Fig. 3:**
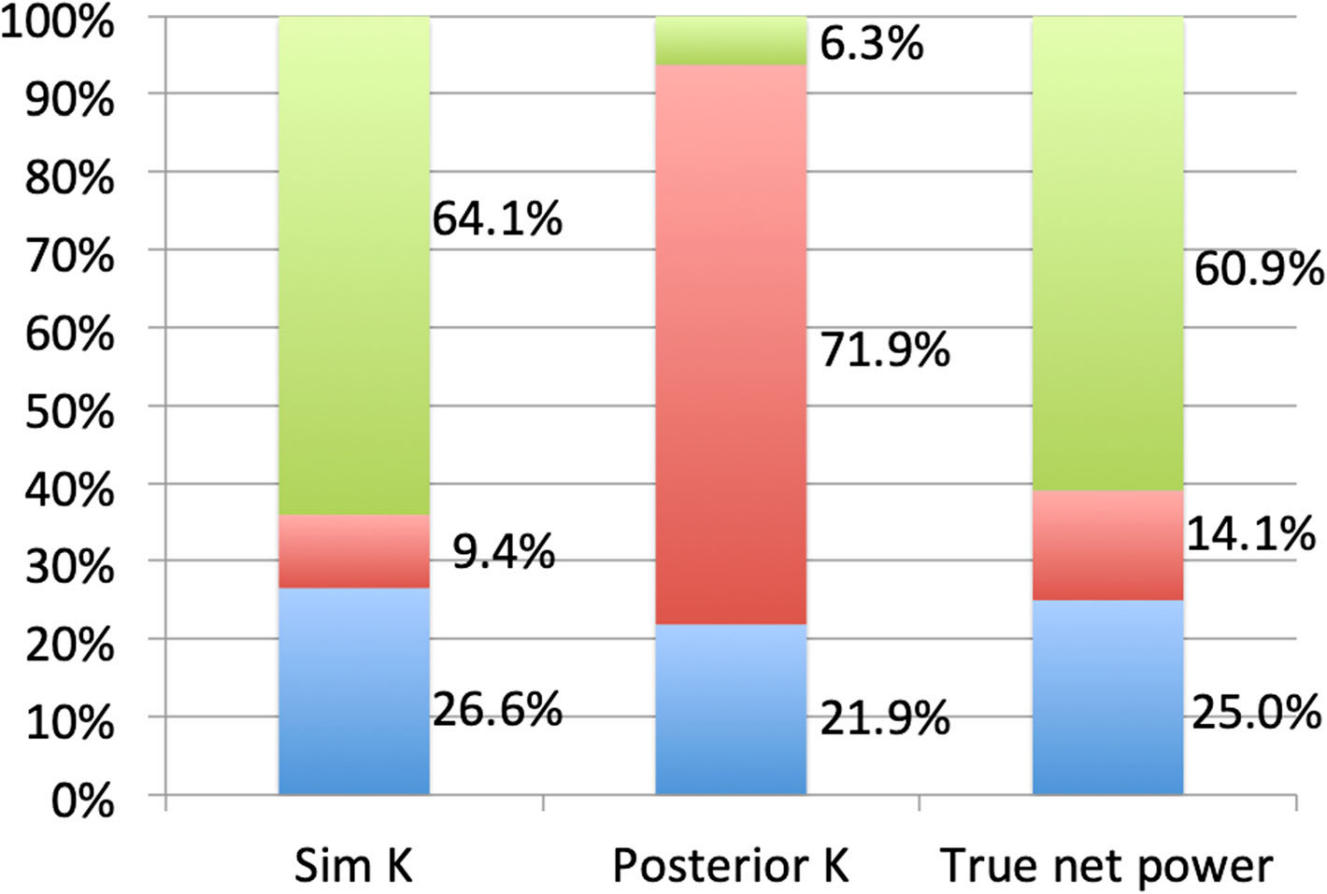
Distributions of corneal astigmatism for simulated K, posterior K, true net power

**Fig. 4:**
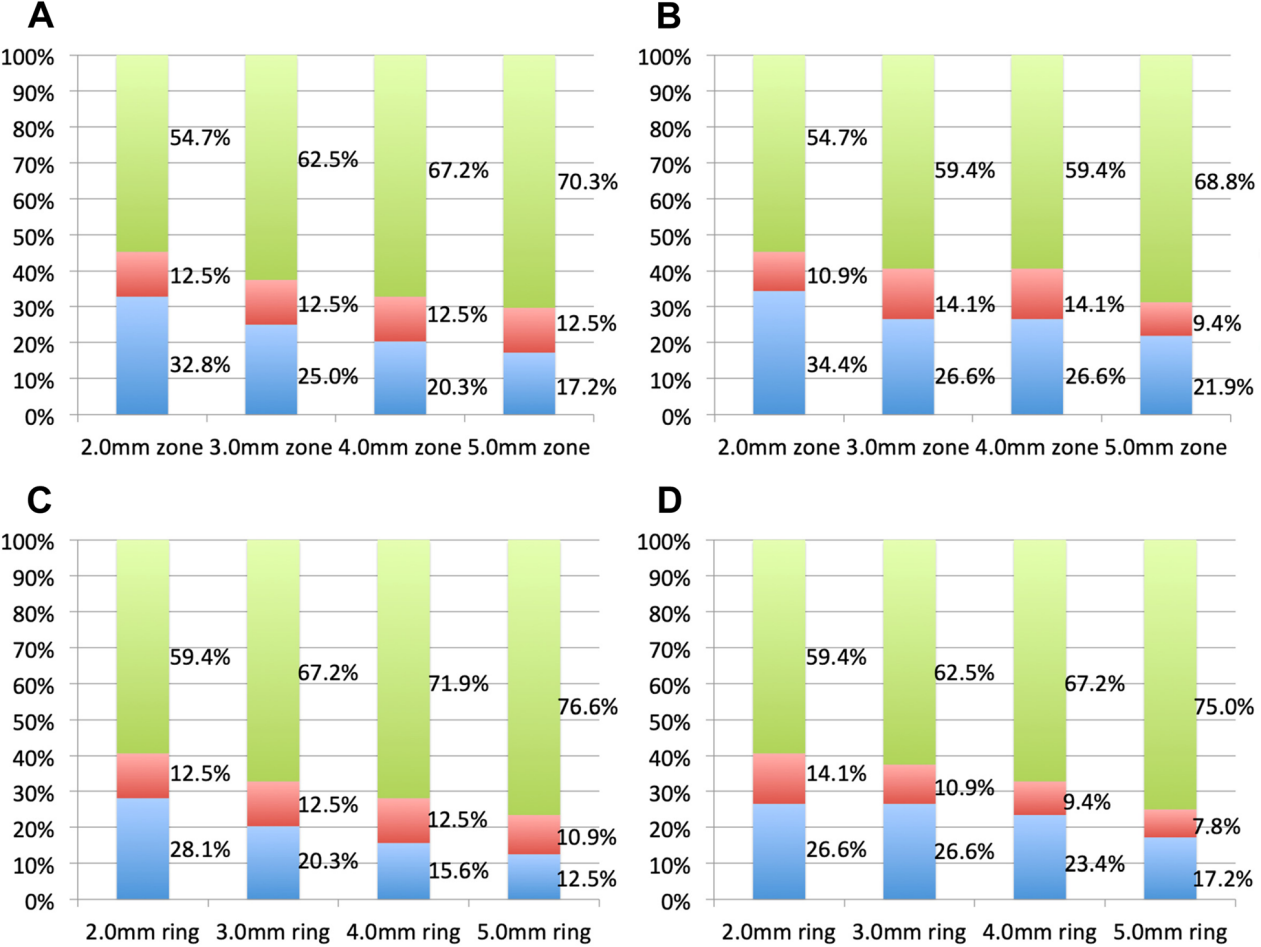
Distributions of corneal astigmatism for total corneal refractive power (TCRP). **a** 2.0 mm–5.0 mm zone centered on the pupil; (**b**) 2.0 mm–5.0 mm zone centered on the apex; (**c**) 2.0 mm–5.0 mm ring centered on the pupil; (**d**) 2.0 mm–5.0 mm ring centered on the apex

Figures. 3 and 4 depict the distribution of corneal astigmatism according to steep meridian. On the anterior corneal surface, the proportion of WTR astigmatism was greatest, followed by oblique, and ATR astigmatism. In contrast, the opposite pattern is observed in the distribution of posterior corneal astigmatism: The proportion of ATR astigmatism was greatest, followed by oblique astigmatism and WTR. As the measurement area increased, the proportion of WTR increased, while the proportion of oblique astigmatism decreased. TCRP centered on pupil resulted in a greater proportion of WTR astigmatism than that centered on the apex.

## Discussion

The present study demonstrated that keratometric measurements including corneal power and astigmatism are influenced by the method used to calculate such values and measurement area. To the best of our knowledge, this study is the first study to evaluate various methods for calculating total corneal power and astigmatism in patients with keratoconus.

TNP values as measured using a Scheimpflug rotating camera in previous studies of the normal cornea were flatter than simulated keratometry (Sim-K) values [22, 23] and these were consistent with the result of the present study. However, some keratometric measurements obtained using a Scheimpflug rotating camera result in greater dioptric powers when compared to simulated keratometry (Sim-K). Corneal power on the flat axis as calculated from EKR and TCRP in the 2.0 mm zone centered on the apex exhibited greater power values than Sim-K. In addition, steep K and mean K values calculated from TCRP at 2.0 mm zone, 3.0 mm zone, and 2.0 mm ring centered on the apex were greater than those obtained from Sim-K.

Calculation of corneal power at more peripheral areas results in lower values for refractive power in patients with keratoconus, opposite to what is observed in the normal cornea. Naeser et al. [24] concluded that TCRP increases with pupil size due to positive spherical aberration and further demonstrated that differences between TCRP values centered on the pupil versus apex ranged from 0.01 to 0.02 diopters in the 2.0 ~ 5.0 mm zones/rings. In the present study, TCRP values centered on the apex were significantly greater than those centered on the pupil with respect to all parameters of corneal power, with the exception of steep keratometry at the 5.0 mm ring. Differences in TCRP ranged from 0.68 diopters (2.0 mm zone) to 1.21 diopters (2.0 mm ring) for flat K; from 0.07 diopters (5.0 mm ring) to 2.41 diopters (2.0 mm zone) for steep K; and from 0.33 diopters (5.0 mm ring) to 1.55 diopters (2.0 mm zone) for mean K.

In the normal cornea, posterior astigmatism is steepest vertically when acting as a minus lens, creating what is known as against-the-rule ocular astigmatism [12]. Ho et al. [12] measured posterior corneal astigmatism and reported that the proportion of against-the-rule (ATR) astigmatism was 96.1% (474 eyes), while the proportion of with-the-rule (WTR) astigmatism was only 2.0% (10 eyes). Koch et al. [12] further concluded that the prevalence of WTR corneal astigmatism has been overestimated and the prevalence of ATR astigmatism has been underestimated. The mean power values for posterior astigmatism as reported by Ho et al. [11], Koch et al. [12], and Zhang et al. [25] are 0.30 D, 0.30 ± 0.15 D, and 0.33 ± 0.16 D, respectively. In eyes with keratoconus, the posterior corneal astigmatism displays large and variable values for total corneal astigmatism [26]. Anterior corneal astigmatism and posterior corneal astigmatism were 4.47 ± 2.05 diopters and 0.87 ± 0.44 diopters in the present study. The mean magnitudes of power were similar to those obtained in prior studies, ranging from 3.05–4.49 diopters for the anterior cornea and from 0.71–0.93 diopters for the posterior cornea [4, 5, 27]. Previous studies have also evaluated the axis orientation of astigmatism [4, 5]. WTR astigmatism is more prevalent on the anterior corneal surface, while ATR astigmatism is more prevalent on the posterior corneal surface in the eyes of Japanese patients with keratoconus [4]. In contrast, ATR astigmatism is more prevalent on the anterior cornea, while WTR astigmatism is more prevalent on the posterior cornea in the eyes of Iranian patients [5]. In the present study, we evaluated the eyes of Korean patients diagnosed with keratoconus and obtained results similar to those of the previous studies, indicating that type of astigmatism may be influenced by ethnicity.

As the measurement zone or ring moved toward the peripheral area, the proportion of WTR astigmatism increased, and mean corneal astigmatism on the double-angle polar plots shifted toward WTR astigmatism. On the other hand, the magnitude of corneal astigmatism exhibited a different pattern of change: The Magnitude of mean corneal astigmatism on double-angle polar plots as determined from TCRP centered on pupil increased with more peripheral measurement zones. In contrast, the magnitude of mean corneal astigmatism as determined from TCRP centered on the apex tended to decrease with more peripheral measurement areas.

We also performed subgroup analysis in this study. For TCRP zone and TCRP ring centered on apex, the mean K did not change according to the measurement area in stage 1 keratoconus. On the other hand, in stage 2 ~ 4 keratoconus, the mean K decreased significantly in refractive dioptric power as the measurement area approached the peripheral area. For mean arithmetic astigmatism, stage 1 keratoconus yielded significant difference in TCRP 2.0 ~ 5.0 mm zone centered on apex and TCRP 2.0 ~ 5.0 mm ring centered on pupil. The above results were also the opposite of those of stage 2–4 keratoconus. These differences due to staging might be related to cone location. When the distance from the maximum K to the apical center provided by the scheimpflug rotating camera was measured, stage 2–4 keratoconus showed a greater value than stage 1 keratoconus [1.88 ± 1.12 mm (range: 0 ~ 4.42 mm) versus 0.93 ± 0.63 mm (range: 0.12 ~ 3.20 mm) / *p* value < 0.001 by Mann-Whitney U test].

There is a limitation in the present study. We did not investigate the best measurements of corneal power in the present study. Further studies evaluating changes in corneal power following corneal cross-linking or intracorneal ring segment implantation are required in order to determine the most appropriate measurements that account for surgically induced refractive change, which may also be helpful in the calculation of intraocular lens (IOL) power calculation and evaluation of disease progression. In addition, finding the best corneal astigmatism reflecting manifest refractive cylinder may be useful in toric IOL implantation.

## Conclusions

In this study, we observed that some total corneal power calculated from measurements obtained from more central areas results in greater corneal refractive power than simulated K. Although changes in the magnitude of corneal astigmatism according to measurement area varied with the method used for the calculation of total corneal, all parameters indicated that more peripheral areas exhibit a higher proportion of WTR astigmatism. We further observed that measurements of TCRP centered on the apex are greater than those centered on the pupil. We believe these findings will help to enhance our understanding of the anatomical and optical characteristics of keratoconus as well as our ability to diagnose keratoconus and determination IOL power in the future.

## Data Availability

The datasets used and/or analysed during the current study available from the corresponding author on reasonable request.
